# Supplementary material for: Predictive Roles of ADAM17 in Patient Survival and Immune Cell Infiltration in Hepatocellular Carcinoma
Source: Int J Mol Sci. 2023 Dec 2;24(23):17069. doi: 10.3390/ijms242317069 (PMC10707406; doi:10.3390/ijms242317069)
Supplement: Supplementary file 1 [file ijms-24-17069-s001.zip › ijms-2725953-supplementary.pdf]

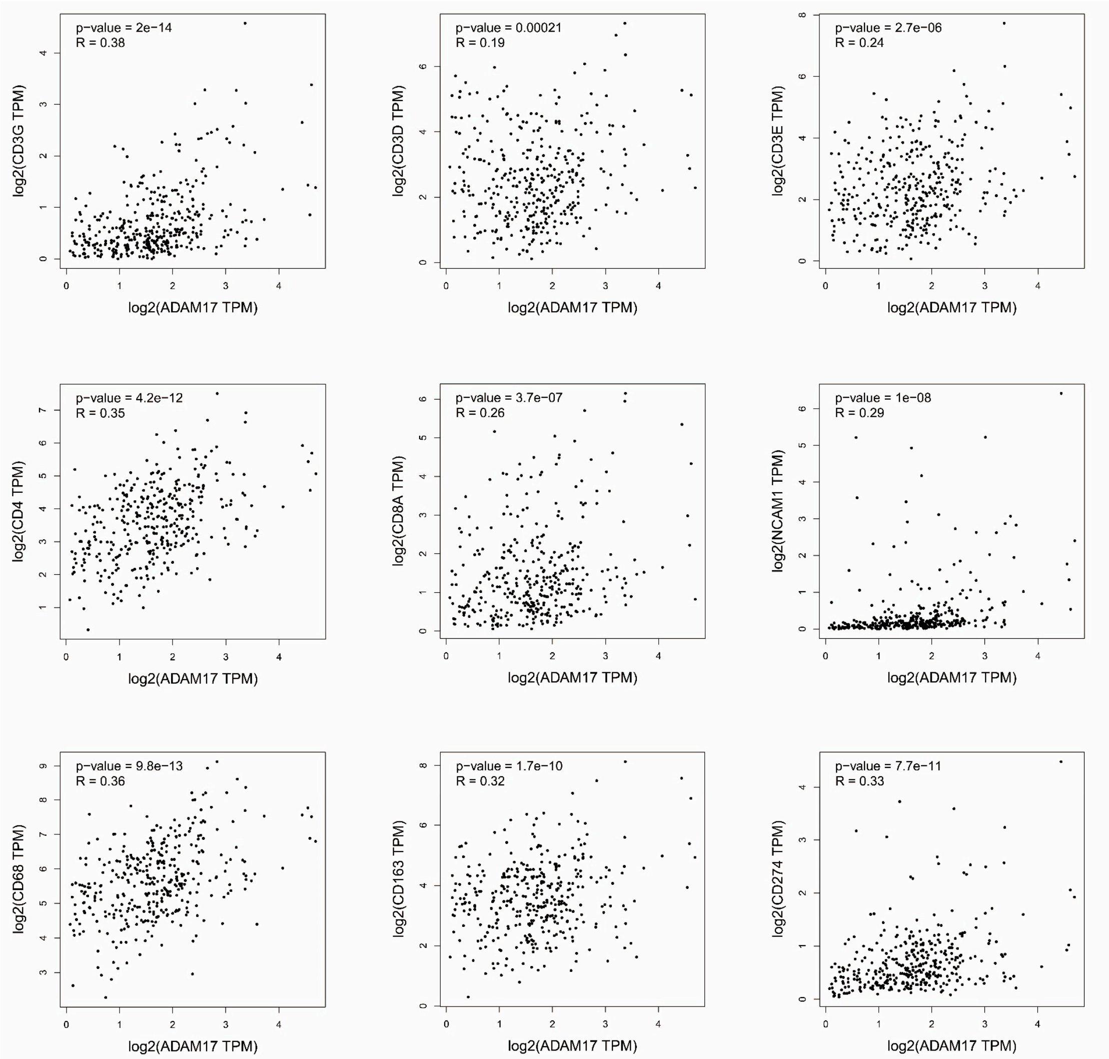

Supplementary Figure S1. Verification for the immunoregulatory roles of ADAM17 in HCC. Correlation analyses of the association between ADAM17 mRNA level and the indicated cell markers using the GEPIA dataset.
